# Early pregnancy body mass index outweighs gestational weight gain in predicting gestational diabetes mellitus: A single-center experience from Türkiye

**DOI:** 10.5339/qmj.2026.53

**Published:** 2026-08-18

**Authors:** Emre Gezer, Özlem Alkan, Yeliz Demirhan, Alev Selek, Berrin Çetinarslan, Zeynep Cantürk, Mehmet Sözen

**Affiliations:** 1 Kocaeli University, Faculty of Medicine, Department of Endocrinology and Metabolism, Kocaeli, Türkiye

**Keywords:** Gestational diabetes, body mass index, pregnancy, weight gain, Türkiye.

## Abstract

**Introduction:**

Gestational diabetes mellitus (GDM) is a common pregnancy complication associated with maternal and neonatal morbidity. Various risk factors for GDM, including pre-pregnancy body mass index (BMI) and gestational weight gain (GWG), have been described in the literature. Although both factors have been identified as important risk factors for GDM, the relative contribution of each to the development of GDM remains controversial. In this retrospective observational study, we aimed to compare the relative impact of early pregnancy BMI and GWG on the risk of developing GDM in a study from a single tertiary center.

**Materials and Methods:**

Our retrospective study analyzed data from pregnant women who were referred to our clinic between 2017 and 2024 and underwent a 75-g oral glucose tolerance test (OGTT) between the 24^th^ and 28^th^ weeks of gestation. Sociodemographic characteristics, plasma glucose levels at 0, 1, and 2 hours during the OGTT, maternal height and self-reported weight at the onset of pregnancy (when informed of the positive pregnancy test), and body weight at the time of the OGTT were retrieved.

**Results:**

Among the 290 pregnant women included in the study, 45 (15.5%) were diagnosed with GDM. In the multivariate logistic regression analysis, advanced maternal age (*p* = 0.034), a prior history of GDM (*p* = 0.043), excessive gestational weight gain (adjusted odds ratio [aOR] = 2.989; *p* = 0.042), and elevated early pregnancy BMI (aOR = 4.317; *p* = 0.025) were identified as independent risk factors for GDM.

**Conclusion:**

The findings of our study showed that pre-pregnancy BMI was independently associated with a higher risk for GDM compared to gestational weight gain. Additionally, advanced age and a history of GDM were independent risk factors for GDM. These results suggest that clinicians should focus on pre-pregnancy BMI assessment and gestational weight monitoring to reduce the risk of GDM.

## 1. INTRODUCTION

Gestational diabetes mellitus (GDM), characterized by glucose intolerance first identified during pregnancy, is linked to immediate and long-term complications in the mother and fetus.^1^ During the perinatal period, GDM increases the risk of preeclampsia, macrosomia, shoulder dystocia, neonatal hypoglycemia, and cesarean deliveries. In the long term, it substantially increases the likelihood of type 2 diabetes and cardiovascular disease in both mothers and children.^1^ Consequently, identifying and managing the risk factors contributing to GDM, particularly those present before and during pregnancy, has become a critical public health priority.

International guidelines, including those from the American Diabetes Association (ADA) and the International Association of the Diabetes and Pregnancy Study Groups (IADPSG), recommend universal screening for GDM using a 75-g oral glucose tolerance test (OGTT) at 24–28 weeks of gestation in women without previously diagnosed diabetes. According to these criteria, GDM is diagnosed when one or more of the following plasma glucose values are met or exceeded: fasting ≥92 mg/dL, 1-hour ≥180 mg/dL, or 2-hour ≥153 mg/dL.^2,3^

In recent years, the amount of weight gained during pregnancy has been identified as a modifiable risk factor of interest. Excessive gestational weight gain (GWG) is common and has been demonstrated to exacerbate insulin resistance, disrupt maternal glucose metabolism, and consequently increase the risk of GDM.^4^ Several observational studies and meta-analyses have reported that excessive GWG is associated with a higher risk of GDM, as well as adverse maternal and neonatal outcomes, including hypertensive disorders of pregnancy, cesarean delivery, and large- for-gestational- age infants^5^^–^^7^ Similarly, previous studies have demonstrated that women who are obese or overweight before pregnancy have a two- to four-fold higher risk of developing GDM compared with those with normal BMI, and that this association remains independent of other maternal factors across different populations.^8^^–^^13^ Within this framework, the present retrospective study, conducted over an 8-year period at a single tertiary center in Türkiye, aimed to compare the effects of early pregnancy BMI and GWG on the risk of GDM among pregnant women who underwent OGTT in order to ascertain which factor exerts a greater influence on the risk of developing GDM.

## 2. MATERIALS AND METHODS

In this retrospective observational study, data from pregnant women who underwent a 75-g oral glucose tolerance test (OGTT) between the 24^th^ and 28^th^ weeks of gestation at the Endocrinology and Metabolism Outpatient Clinic, a single tertiary center, between January 2017 and December 2024. All data were obtained from the hospital electronic health record system, which was updated in January 2017. No direct contact with patients was involved in the data collection process. The study included all consecutive eligible patients who met inclusion criteria during the study period. No sampling or selection beyond inclusion criteria as described below was applied. For each participant, the following information was recorded: sociodemographic characteristics; personal history of previous GDM diagnosis and family history of diabetes mellitus; parity; plasma glucose levels at 0, 1, and 2 hours during the OGTT; maternal height and self-reported weight at the onset of pregnancy (when informed of the positive pregnancy test); and body weight at the time of the OGTT. GDM is diagnosed if one or more of the following plasma glucose values are met or exceeded during a 75-g OGTT: fasting glucose ≥ 92 mg/dL, 1-hour glucose ≥ 180 mg/dL, or 2-hour glucose ≥ 153 mg/dL (3). GWG was calculated as the difference between the weight measured during the OGTT and the weight at early pregnancy. The body mass index (BMI) was determined using the formula: weight (kg) / height (m)^2^. The individuals were also asked about their physical activity habits.

Women were eligible for inclusion in the study if they were aged ≥ 18 years and had completed a 75-g OGTT. No upper age limit was applied as an exclusion criterion. The exclusion criteria encompassed individuals with a prior diagnosis of type 2 diabetes mellitus who were currently under non-pharmacological management, those using regular medications for chronic conditions such as hypertension prior to pregnancy, and those with a history of bariatric surgery for obesity management. All data were documented in a standardized case report form.

Statistical analyses were performed using IBM SPSS Statistics version 29.0 (IBM Corp., Armonk, NY, USA). The distribution of continuous variables was assessed for normality using the Kolmogorov–Smirnov test. Continuous variables with a normal distribution were presented as mean ± standard deviation, while those not normally distributed were expressed as median (25^th^–75^th^ percentile). Categorical variables were reported as frequencies and percentages. Group comparisons of continuous variables were conducted using the independent samples t-test for normally distributed data or the Mann–Whitney U test for non-normally distributed data. The relationships between categorical variables were evaluated using the chi-square test. Logistic regression modeling was used for multivariate analyses. A *p*-value of <0.05 was considered statistically significant. Ethics approval was obtained from the ethics committee of our institution (Date: 24 April 2025, No: xxx-2025/09/22, project No:2025/207). Informed consent was not required given the retrospective design.

## 3. RESULTS

Out of around 2000 patient records reviewed, only 290 were selected for the study, primarily due to incomplete patient data. Among the 290 pregnant women included in the study, 45 (15.5%) were diagnosed with GDM, while 245 (84.5%) did not develop GDM. The mean age of the participants was 30.1 ± 4.9 years. The demographic and clinical characteristics of the pregnant women are presented in Table 1.

In the univariate analyses, women with GDM were significantly older than those in the non-GDM group (median age 32 vs. 29 years, *p* = 0.002). Furthermore, no statistically significant differences were observed between the groups in terms of educational status, employment, active smoking status, or physical activity levels (Table 2). Sociodemographic factors, such as lower monthly income and family history of diabetes mellitus, were significantly more prevalent in the GDM group (*p* = 0.048 and *p* = 0.010, respectively). Additionally, a history of GDM was more common among women who developed GDM during the current pregnancy (*p* = 0.031).

In relation to anthropometric and gestational weight parameters, both early pregnancy BMI and GWG were significantly associated with gestational diabetes mellitus GDM (Table 3). The incidence of GDM increased progressively across higher BMI categories: 6.4% in women with a BMI of < 25 kg/m², 25.4% in those with a BMI of 25–29.9 kg/m², and 27.0% in those with a BMI of ≥ 30 kg/m² (*p* < 0.001). Furthermore, women who experienced excessive GWG (>7 kg) exhibited a significantly higher prevalence of GDM compared to those with restricted weight gain (22.2% vs. 10.4%, *p* = 0.009). Although a significant association was identified using numerical data concerning GWG, categorical data were employed to increase the statistical significance by using a median value of 7 kg.

In the multivariate logistic regression analysis presented in Table 4, several independent predictors of gestational diabetes mellitus GDM were identified. These predictors included advanced maternal age (adjusted odds ratio [aOR] = 1.126; 95% confidence interval [CI]: 1.009–1.257; *p* = 0.034), a prior history of GDM (aOR = 3.525; 95% CI: 1.039–11.963; *p* = 0.043), excessive (> 7 kg) gestational weight gain (aOR = 2.989; 95% CI: 1.041–8.581; *p* = 0.042), and elevated early pregnancy BMI. Specifically, compared with women with a BMI of < 25 kg/m², those with a BMI ranging from 25 to 29.9 kg/m² exhibited an aOR of 4.317 (95% CI: 1.201–15.522; *p* = 0.025), while those with a BMI of ≥ 30 kg/m² demonstrated an aOR of 4.007 (95% CI: 1.145–14.029; *p* = 0.030).

These results reveal that an increased BMI during early pregnancy and excessive weight gain throughout gestation are independently linked to a heightened risk of GDM. The initial BMI at the onset of pregnancy exerted a greater influence than the weight gain experienced during pregnancy, as indicated by the adjusted odds ratios (aOR = 4.3 vs. 3.0).

## 4. DISCUSSION

To the best of our knowledge, this is the first study from Türkiye evaluating the association between early pregnancy BMI, gestational weight gain, and the risk of GDM. Excess body weight before and during pregnancy has been associated with increased insulin resistance and disturbances in glucose metabolism, which may contribute to a higher risk of gestational diabetes mellitus.^1,2^ Previous studies have similarly reported that elevated BMI and excessive gestational weight gain are associated with a higher risk of GDM.^3,4^ In line with these findings, our study demonstrated that early pregnancy BMI, excessive gestational weight gain, previous GDM history, and older age were independent risk factors for GDM, indicating that increases in these variables were associated with a higher likelihood of developing GDM, while active smoking, presence of 1st degree relative with DM, parity, and physical activity were not significantly associated with the risk of GDM. In a similar study conducted in Oman, Abu-Heija et al.^14^ reported that pre-pregnancy BMI had a significant impact on GDM risk, with higher BMI levels associated with an increased risk of GDM. Another study conducted in Taiwan, including 512 pregnant women, demonstrated that pre-pregnancy BMI was a risk factor for GDM, showing that overweight and obese women were more likely to develop GDM than normal-weight women.^13^ In line with the results of these single-center studies, both a cross-sectional large-scale study and a meta-analysis reported that pre-pregnancy BMI was an independent risk factor for GDM, with progressively increasing BMI categories associated with incrementally higher odds of GDM.^8,10^

In contrast to the significant effect of pre-pregnancy BMI on GDM risk, there are conflicting data on the effect of gestational weight gain in the literature. In a study that evaluated 41,845 pregnant women, the authors reported that excessive GWG in the normal-weight subgroup was associated with a lower GDM risk, which was possibly attributed to confounding factors.^15^ Another study reported by MacDonald et al.^16^ indicated that excessive GWG in the first trimester was significantly associated with GDM, whereas GWG in the second trimester did not affect GDM risk.^16^ In line with this finding, Hedderson et al.^5^ also reported that greater GWG, particularly in the first trimester, was an independent risk factor for GDM, meaning that women with higher early-pregnancy weight gain had higher odds of developing GDM, even after adjusting for pre-pregnancy BMI. Regrettably, in our study, GWG was calculated from the onset of pregnancy until the OGTT was performed. This period was not segmented into two trimesters, precluding a direct comparison of our study outcomes with those of the referenced study. Similar to our findings, a study evaluating more than 8000 pregnant women demonstrated that excessive GWG in the first two trimesters was a significant risk factor for GDM.^17^

Despite various studies investigating the effects of pre-pregnancy BMI and GWG on GDM risk, only a few studies have reported multivariate logistic regression models that included both parameters and provided adjusted odds ratios for both pre-pregnancy BMI and GWG, similar to our study.^18,19^ In one of these studies, data from 1951 pregnant women from Malaysia were evaluated. Elevated early pregnancy BMI (≥25) was associated with a 1.44 times the risk of GDM (*p* = 0.02), while excessive GWG during the first and second trimesters was linked to a 1.01- and 1.17-fold increase in the risk of GDM, respectively, although these associations did not reach statistical significance (*p* = 0.97 and *p* = 0.37, respectively).^18^ In our study, the aORs were higher, indicating a stronger association between both elevated BMI and excessive GWG and GDM risk, which might be due to the major differences between the adjusted factors included in our study, such as previous GDM, active smoking, physical exercise status, and family history of DM, which were not included in the referenced study. In a case-control study from China, which included fewer pregnant women (90 GDM cases and 165 non-GDM control group) and fewer adjusted factors than our study, the researchers showed that higher pre-pregnancy BMI (≥24) and GWG were associated with a 2.58-fold and 2.03-fold higher risk for GDM, respectively.^19^ In both studies, the effects of pre-pregnancy BMI on GDM were greater than those of GWG, which is consistent with our findings. However, the aORs in our study were higher than those reported in these cited studies, which might be attributed to the inclusion of more confounding factors in our multiple logistic regression model.^18,19^

In this study, other findings, such as a significant correlation between advanced age and previous GDM history and GDM risk, were consistent with the literature.^10,14,20,21^ In addition, our finding of a nonsignificant relationship between active smoking and GDM risk is also in line with two meta-analyses from Asia and Europe.^22,23^ Prenatal smoking history has been shown to have a significant impact on the development of GDM, possibly by triggering inflammatory responses, oxidative stress, and insulin resistance.^24^^–^^26^

In contrast to the literature, we found that parity, physical exercise, and family history of DM were not independent risk factors for GDM. In a recent comprehensive meta-analysis, a family history of DM increased the risk of GDM by 1.85 times.^20^ A review article stated that individuals with an increased BMI who engage in physical activity during pregnancy experience an approximately 50% reduction in the risk of developing GDM compared to their sedentary counterparts.^4^ The observed discrepancy between our findings and those reported in the literature may be attributed to a limitation inherent in our study, specifically, the reliance on self-reported data. Cultural differences in the perception of physical exercise may account for this variation. For instance, activities such as walking to the market or house cleaning may be perceived as physical exercises by some individuals in Türkiye; however, these activities may also be considered part of normal daily life rather than intentional physical exercise. Therefore, self-reported physical exercise status may not be a reliable parameter. In a review by Dode et al.^27^ higher parity was reported to be associated with an increased risk of GDM; however, this association was largely influenced by maternal age and obesity, which act as important confounding factors. Surprisingly, in line with our findings, two studies reported that parity was not a significant independent risk factor for GDM.^21,28^

The main strengths of this study include its focus on early pregnancy BMI and gestational weight gain within the same multivariate model, as well as the use of real-world clinical data. However, as this was a single-center study conducted at a tertiary care institution, the generalizability of the findings may be limited. In addition, this research is primarily limited by its retrospective nature. Despite screening around 2,000 patients, only 290 were eligible for inclusion due to incomplete or missing clinical data necessary for analysis. This significant reduction in sample size may have led to selection bias and restricts the generalizability of the results. Furthermore, the dependence on existing medical records limited the ability to control data quality and standardize variables.

## 5. CONCLUSION

The findings of our study showed that pre-pregnancy BMI was independently associated with a higher risk for GDM compared to gestational weight gain. Additionally, advanced age and a history of GDM were independent risk factors for GDM, whereas active smoking, parity, and family history of DM had no significant impact on the development of GDM. To date, only a few studies have reported data on the adjusted odds ratios of pre-pregnancy BMI and GWG, analyzed in the same multivariate logistic regression model. These results suggest that clinicians should focus on pre-pregnancy BMI assessment and gestational weight monitoring to reduce the risk of GDM. Furthermore, to better identify independent risk factors for GDM within the Turkish population, large-scale retrospective observational studies, including nationwide data from all regions, are warranted.

## ACKNOWLEDGEMENTS

We would like to thank Assoc. Prof. Sibel Balci, from the Department of Biostatistics, Faculty of Medicine, Kocaeli University, for helping us perform statistical analyses of our data.

## AUTHORS’ CONTRIBUTIONS

EG, ÖA, and AS: Concept and design. EG and AS: Methodology. YD. and ÖA: Data collection. YD, EG, and MS: Statistical analysis. EG and ÖA: Writing – original draft. BÇ and ZC: Writing – review and editing. BÇ, ZC, and AS: Supervision.

## CONFLICT OF INTERESTS

The authors declare that they have no conflicts of interest.

## DISCLOSURE OF AI USE

OpenAI (ChatGPT) tool was used solely for English language editing and grammar checking. All revisions were reviewed and approved by the authors.

## DATA AVAILABILITY

The data supporting the findings of this study can be obtained from the corresponding author upon reasonable request.

## FUNDING

The authors did not receive any financial support for the research, authorship, or publication of this article.

## ETHICAL APPROVAL

Ethics approval was obtained from the Ethics Committee of Kocaeli University, Faculty of Medicine (No: KU GOKAEK- 2025/09/22, project number: 2025/207).

## Figures and Tables

**Table 1.** Demographic and clinical characteristics of pregnant women undergoing OGTT at a single tertiary center in Türkiye (2017-2024) (*n* = 290).

	Category	n (%)
Age (years) (Mean ± SD)		30.1 ± 4.9
GDM	Yes	45 (15.5)
	No	245 (84.5)
Educational status	Illiterate	10 (3.6)
	Primary school	23 (8.2)
	Middle school	49 (17.6)
	High school	73 (26.2)
	University	113 (40.5)
	Postgraduate	11 (3.9)
Employment status	Housewife	135 (47.2)
	Employed	151 (52.8)
Monthly income	<10,000 TL	35 (12.1)
	10,000–20,000 TL	78 (27.0)
	20,000–30,000 TL	90 (31.1)
	>30,000 TL	86 (29.8)
Smoking	Yes	36 (12.4)
	No	254 (87.6)
Multiparity (≥2)	Yes	150 (51.9)
	No	139 (48.1)
Previous GDM	Yes	22 (15.4)
	No	121 (84.6)
First-degree relative with DM	Yes	122 (43.6)
	No	158 (56.4)
Physical activity	Mostly sedentary	13 (4.6)
	Low daily activity	88 (31.0)
	Active and mobile	183 (64.4)

DM: Diabetes mellitus, GDM: Gestational diabetes mellitus, TL: Turkish lira.

**Table 2.** Comparison of sociodemographic and clinical variables between GDM and non-GDM groups at a single tertiary center in Türkiye (2017–2024).

	Category	GDM (*n* = 45)	Non-GDM (*n* = 245)	*p*-value*
Age (years)^α^		32 (28-35)	29 (27-33)	**0.002** ^β^
Educational Status	Illiterate	3 (30.0%)	7 (70.0%)	0.188
	Primary school	7 (30.4%)	16 (69.6%)	
	Middle school	7 (14.3%)	42 (85.7%)	
	High school	13 (17.8%)	60 (82.2%)	
	University	13 (11.5%)	100 (88.5%)	
	Postgraduate	1 (9.1%)	10 (90.9%)	
Employment status	Housewife	27 (20.0%)	108 (80.0%)	0.061
	Employed	18 (11.9%)	133 (88.1%)	
Monthly income	<10,000 TL	9 (25.7%)	26 (74.3%)	**0.048**
	10,000–20,000 TL	16 (20.5%)	62 (79.5%)	
	20,000–30,000 TL	13 (14.4%)	77 (85.6%)	
	>30,000 TL	7 (8.1%)	79 (91.9%)	
Smoking	Yes	6 (13.3%)	30 (12.2%)	0.839
	No	39 (86.7%)	215 (87.8%)	
Multiparity	Yes	28 (62.2%)	122 (50.0%)	0.132
	No	17 (37.8%)	122 (50.0%)	
Previous GDM	Yes	8 (36.4%)	14 (63.6%)	**0.031**
	No	18 (14.9%)	103 (85.1%)	
1st-degree Relative with DM	Yes	27 (22.1%)	95 (77.9%)	**0.010**
	No	17 (10.8%)	141 (89.2%)	
Physical activity	Sedentary	2 (15.4%)	11 (84.6%)	0.411
	Low activity	17 (19.3%)	71 (80.7%)	
	Active	24 (13.1%)	159 (86.9%)	

DM: Diabetes mellitus, GDM: Gestational diabetes mellitus, TL: Turkish lira. *p*-values in bold are statistically significant.

*Data were analyzed using the Chi-Square test. ^α^Data are expressed as median and interquartile range. ^β^Data were analyzed using the Mann-Whitney U test.

**Table 3.** Association between early pregnancy BMI / gestational weight gain and GDM at a single tertiary center in Türkiye (2017–2024).

	Category	GDM (*n* = 45)	Non-GDM (*n* = 245)	*p*-value*
BMI at Beginning of Pregnancy, *n* (%)	<25	10 (6.4%)	146 (93.6%)	**<0.001**
	25–29.9	18 (25.4%)	53 (74.6%)	
	≥30	17 (27.0%)	46 (73.0%)	
Gestational Weight Gain, *n* (%)	Restricted^α^	17 (10.4%)	147 (89.6%)	**0.009**
	Excessive^β^	28 (22.2%)	98 (77.8%)	

BMI: Body mass index, DM: Diabetes mellitus, GDM: Gestational diabetes mellitus. *p*-values in bold are statistically significant.

*Data were analyzed using the Chi-Square test. ^α^Restricted gestational weight gain, defined as ≤7 kg (median). ^β^Excessive gestational weight gain, defined as >7 kg (median).

**Table 4.** Multivariate logistic regression analysis for GDM in pregnant women at a single tertiary center in Türkiye (2017–2024).

	aOR	95% CI for OR	*p*-value
1^st^-degree relative with DM	1.677	0.604 – 4.661	0.321
Active smoking	1.047	0.239 – 4.588	0.951
Age	1.126	1.009 – 1.257	**0.034**
Multiparity	0.249	0.034 – 1.845	0.174
Previous GDM	3.525	1.039 – 11.963	**0.043**
Physical activity (R: active)			0.849
- Low activity	1.126	0.391 – 3.241	0.825
- Sedentary	0.551	0.050 – 6.067	0.626
Gestational weight gain >7 kg	2.989	1.041 – 8.581	**0.042**
BMI at beginning of pregnancy (R: <25)			**0.040**
- 25–29.9	4.317	1.201 – 15.522	**0.025**
- ≥30	4.007	1.145 – 14.029	**0.030**

aOR: Adjusted odds ratio, BMI: Body mass index, CI: Confidence interval, DM: Diabetes mellitus, DM: Gestational diabetes mellitus, OR: Odds ratio, R: Reference category.

*p*-values in bold are statistically significant.
